# The psychological health of forensic investigators following mass fatality incidents: a cross-sectional study 9 months after the Jeju Air crash in South Korea

**DOI:** 10.3389/fpubh.2026.1819655

**Published:** 2026-05-29

**Authors:** Euntaek Hong, Subin Park, Gaeun Son, Haeyoun Choi, Kee-Hong Choi

**Affiliations:** 1 School of Psychology, Korea University, Seoul, Republic of Korea; 2 Department of Psychology, Chungbuk National University, Cheongju, Republic of Korea; 3 Korea University Mind Health Institute, Seoul, Republic of Korea

**Keywords:** first responder, forensic investigator, mass fatality incident, mental health, PTSD, trauma-informed care

## Abstract

**Introduction:**

Following the Jeju Air crash in Muan (South Korea) on December 29, 2024, resulting in 179 fatalities, forensic investigators (FIs) were exposed to traumatic scenes while performing disaster victim identification (DVI) procedures. FIs are known to be at significant risk for post-traumatic stress disorder (PTSD) and other mental health issues due to their routine exposure to traumatic scenes. Despite its clinical significance, there remains a paucity of empirical research quantitatively evaluating the psychological ramifications of DVI procedures and associated occupational stressors on FIs.

**Methods:**

Therefore, the current cross-sectional survey was conducted among a total of 735 FIs, including 224 individuals who had been engaged in the Jeju Air crash site 9 months prior to the study. Mental health outcomes, including probable PTSD (IES-R-K defined), depression (PHQ-9 defined), anxiety (GAD-7 defined), alcohol use (AUDIT-C defined), and suicide risk (MHS: S defined), were evaluated. We used Chi-square tests to compare symptom prevalence between deployed and non-deployed FIs, and applied correlation and adjusted logistic regression analyses to identify MFI site-specific factors associated with probable PTSD.

**Results:**

Within the entire sample, the prevalence of probable PTSD and overall mental disorders reached 10.2% [95% CI: (8.0, 12.4)] and 19.7% [95% CI: (16.9, 22.6)]. Engaged FIs experienced significantly more frequent traumatic events and a higher probable PTSD prevalence [15.2, 95% CI: (10.5, 19.9)]. Traumatic experiences on scenes during DVI procedures, identification-related distress, and adverse working conditions were identified as factors significantly associated with probable PTSD.

**Discussion:**

These results offer critical insights into the mental health status and related site-specific factors in disaster contexts. The study supports the implementation of trauma-informed and evidence-based mental health initiatives to protect FIs exposed to MFIs.

## 1 Introduction

On December 29, 2024, Jeju Air Flight 2,216 crashed into a concrete embankment while attempting an emergency landing at Muan International Airport in South Korea, resulting in 179 fatalities out of 181 passengers. A group of 373 forensic investigators (FIs) were dispatched to perform forensic tasks according to the disaster victim identification (DVI) process. They managed to complete all recovery processes in 9 days, but had to struggle with exposure to severely damaged remains, lack of sleep, excessive workload, and pressures of making mistakes (1).

First responders (FRs) in a mass fatality incident (MFI) are faced with various stressors, resulting in a high prevalence of mental health disorders. While responding to catastrophic and large-scale MFIs, FRs are constantly exposed to psychologically traumatic events such as serious injuries or deaths and being distressed by strenuous working conditions, all of which are significant risk factors of post-traumatic stress disorder (PTSD) (2–5). The prevalence of PTSD among FRs responding to MFIs was reported from 10.2 to 16.9% (6–8), which is higher than the trauma-exposed population prevalence rates of PTSD (5.6%) (9). The psychological vulnerability often extends to comorbid mental health conditions such as depression, anxiety, and substance use (2, 6, 8, 10).

Among FRs, FIs who play the specialized role in forensic scenes are especially vulnerable to various traumatic events and stressors while recovering and identifying evidence. Usually working in isolation, FIs have to directly handle the damaged human remains while leaning heavily on the sensory cues (e.g., morphology, thermal damage, and olfactory imprints) (11). The tasks of FIs require repeatedly revisiting and investigating the same scenes and remains, which may invoke vicarious trauma (VT), psychological distress from persistent exposure to traumatic events (12, 13). These indirect and routine traumatic exposures during duty often lead FIs to high risk of PTSD, with prevalence of 9.3–21.6% (14, 15), also higher than that of general trauma-exposed populations.

In response to MFIs, the risk of mental health among these personnel increases. Brondolo and colleagues elucidated the three main PTSD triggers of FIs dispatched to MFI sites (16); they cannot expect the details about the timing, nature, and subsequent caseload of the disaster in advance (unpredictability), but have to encounter unmanageable emotional experiences while providing support to the victims and their families (uncontrollability), are therefore overwhelmed by emotional harm and secondary stressors (threat), resulting in psychological vulnerability of FIs.

In addition to the exposure to mass casualties, witnessing bereaved families overwhelmed by intense grief and anger on MFI sites is likely one of the most unpredictable, uncontrollable, and threatening experiences for FIs, who typically handle remains alone and hardly contact survivors in person. FRs who repeatedly interacted with victims or the bereaved appeared to be vulnerable to compassion fatigue due to empathy-based stress, and moral injury when their occupational belief that they must protect and take responsibility for the safety of the victims was shattered, all of which are discrete risk factors of mental health (17). Such unexpected encounters can trigger emotional identification with victims, feelings of “It could have been my friend,” or “It could have been me” especially when the characteristics (e.g., appearance, age, job) of the victim are similar to one’s self or one’s family or friends (18). This experience of identification was known to significantly predict symptoms of PTSD, such as intrusive and avoidant symptoms (5). Furthermore, the large scale of MFIs often leads to a strenuous operational environment, which triggers chronic overwork, sleep deprivation, and food shortages (19). This threatening work-related distress appears to deplete FIs’ physical and psychological resources, thereby increasing the risk of developing problematic mental health conditions. However, FIs are generally not prepared enough to manage their psychological reactions in front of mass casualties and provided with proper support before and during incidents, thereby increasing their mental health vulnerability (20).

Although there have been several studies identifying mental health vulnerability of FIs in general as mentioned earlier, few studies focus on unique patterns of exposure and occupational stressors among FIs and their relationship with mental health (21). Furthermore, to our knowledge, no studies have empirically investigated the mental health status and stressors associated with mental health of FIs deployed in the unique context of MFIs. These research gaps lead to a lack of psychological support for FIs, which negatively affects both the individual and organization. Untreated psychological trauma impairs work efficiency, potentially increasing sick leave or resignation of FIs (22). The culture of neglecting mental health problems does not only end up with decline in their quality of life but also occupational investigative outcomes and integrity, resulting in loss of public trust in the justice system (23). Therefore, it is imperative to identify the mental health conditions and their relationship with work-related experience of FIs, especially those deployed in MFIs.

The primary objective of this study is to empirically evaluate the mental health status of overall FIs and FIs who participated in recovery of the Jeju Air crash 9 months after the incident. This research has three specific aims:

1. To measure the prevalence and severity of probable PTSD and comorbid psychological health issues (depression, anxiety, suicidal risk, and alcohol use) among the overall FI sample.
2. To compare the mental health prevalences among the engaged FI sample with those of a control group of non-deployed FI sample.
3. To identify DVI-specific duty types and stressors that were significantly associated with probable PTSD.

## 2 Methods

### 2.1 Procedure

Data collection was conducted via an online survey from September 11th to October 2nd, 2025. The recruitment was posted on the intranet of the National Office of Investigation, ensuring all participants serve as FIs in South Korea. All participants provided informed consent to participate in the study. The present study was approved by the local review board. The reporting of this study was conceptualized and updated following the STROBE guidelines (Supplementary material) (24).

### 2.2 Measures

#### 2.2.1 Demographic and work-related information

Participants provided demographic information including gender, age, marital status, and household characteristics. To evaluate professional differences, the questionnaire also assessed work-related variables such as employment type, nature of duty, agency affiliation, rank, length of service in both the general police force and forensic investigation, and the number of exposures to severely damaged and massive bodies before Jeju Air crash.

#### 2.2.2 Duty types and stressors at site

The study assessed duty types and stressors of FIs who directly or indirectly engaged in the Jeju Air crash response (*n* = 224). To evaluate the relationship between psychological health and specific duties, we expanded the standard four-phase DVI procedure—scene (recovery), ante-mortem (collection of samples from bereaved family members), post-mortem (autopsy and body reconstruction), and reconciliation—to include administrative support and the release of remains to families, as these tasks were prominent in this incident while duties of responders were intermingled. Participants rated their distress for each duty using a 4-point Likert scale.

Based on prior research (25) and the preliminary interviews (26), we also specified ten stress-inducing events, ranging from the handling of infant remains to perceived pressure regarding identification errors. The frequency and subjective distress of these stressors were measured consistently with the duty type assessment.

For the logistic regression, we dichotomously coded responses as 1 if participants reported experiencing the corresponding duty type or stressor, and as 0 if they did not experience it or did not participate in the work (non-deployed FIs, *n* = 511).

#### 2.2.3 Psychological health

Due to the nature of the online survey, we used self-report screening tools, rather than diagnostic instruments, to measure probable PTSD and overall mental health of FIs.

##### 2.2.3.1 PTSD

The Impact of Event Scale is a 22-item measurement with 5-point Likert scale, which measures how much the potentially traumatic event impacts individual’s mental health. The scale was developed by Horowitz et al. (27) and revised by Weiss and Marmar (28). The present study used the Korean-validated version of the Impact of Event Scale-Revised (IES-R-K) (29). In order to align more strictly with Criterion A of the DSM-5-TR for a clinical PTSD diagnosis (exposed to death, threatened death, actual or threatened serious injury, or actual or threatened sexual violence, including indirect exposure to aversive details of the trauma, usually in the course of professional duties), we asked participants to specify the “single most distressing event while working”. We classified participants as having probable PTSD only if they both indicated a traumatic event and exceeded the IES-R-K cutoff point (25 or over). The internal consistency of IES-R-K in this study was found to be excellent (Cronbach’s alpha: 0.96).

##### 2.2.3.2 Depression

The Patient Health Questionnaire-9 (PHQ-9) (30) is a 9-item measurement with a 4-point Likert scale, which ranges from 0 to 3. The PHQ-9 is used to examine the severity of depressive symptoms within past 2 weeks. Total scores of 5–9, 10–19, and 20 and over indicate mild, moderate, and severe depressive symptoms, respectively. A total score of 10 (moderate or severe) is the cutoff for the clinical level of depression. We used the Korean version of the PHQ-9, validated by Park et al. (31). Its internal consistency in this study was excellent (Cronbach’s alpha: 0.90).

##### 2.2.3.3 Anxiety

The General Anxiety Disorder-7 (GAD-7) (32) is a 7-item measurement with a 4-point Likert scale, which ranges from 0 to 3. The GAD-7 is used to examine the severity of anxious symptoms within past 2 weeks. We used the Korean version of the GAD-7, validated by Seo et al. (33). Total scores of 5–9, 10–19, and 20 and over indicate mild, moderate, and severe levels of anxious symptoms, respectively. A total score of 10 (moderate or severe) is the cutoff for the clinical level of anxiety. Its internal consistency in this study was excellent (Cronbach’s alpha: 0.92).

##### 2.2.3.4 Suicide risk

The Mental Health Screening Tool for Suicide Risk (MHS:S) (34) is a 4-item questionnaire with a 5-point Likert scale, which ranges from 0 (Never) to 4 (Always). The MHS:S was used to measure the suicide risk, developed and validated in Korean. Total scores of 1, 2, and 3 indicate mild, moderate, and severe suicidality, respectively. A total score of 2 or over is classified as having the clinical level of suicide risk. Its internal consistency in this study was good (Cronbach’s alpha: 0.88).

##### 2.2.3.5 Alcohol use

The Alcohol Use Disorders Identification Test-Consumption (AUDIT-C) (35) is a three-item version of AUDIT (36), which is focused on quantity and frequency of alcohol consumption. We used the Korean version of the AUDIT-C, in which the total score of 8 or over indicates heavy alcohol consumption according to the validation study for Korean population (37). Its internal consistency in this study was good (Cronbach’s alpha: 0.84).

### 2.3 Data collection

Data collection was conducted anonymously via the online survey platform Qualtrics. Of the total of 1,101 records, valid responses of 735 participants were analyzed after elimination of the uncompleted or random responses filtered by time stamps and response patterns.

### 2.4 Statistical analysis

Descriptive statistics, including means, standard deviations, and frequencies, were calculated to characterize the study sample in terms of demographics, duty types, stressors, and mental health scores. To examine associations between engaged FI experience and clinical outcomes—specifically probable PTSD, depression, anxiety, suicide risk, and alcohol use—two-tailed Chi-square tests were performed. For significant results, post-hoc analyses using standardized residuals were conducted.

We used Spearman’s correlation analysis to explore correlations between PTSD symptom scores (avoidance, hyperarousal, intrusion, numbing, total) and duty roles or stressors. The significance level (0.05) was adjusted for using the Bonferroni correction (5 symptom scores * 17 roles & stressors = 85 tests; *α* = 0.05/85 = 0.00059).

Logistic regression was used to identify duty types and stressors associated with probable PTSD controlling for baseline characteristics (demographic and previous work-related variables) and all MFI-related variables (fully adjusted models). A fully adjusted duty type model had 12 predictors, and a fully adjusted stressor model had 15 predictors. All models included with 75 probable PTSD cases and 660 FIs without probable PTSD (*N* = 735). The variance inflation factors (VIF) for variables included in all fully adjusted models were found to be <10, indicating that no serious multicollinearity issues—which can arise from high correlations among independent variables when multiple variables are included simultaneously (38)—were observed. However, some duty type and stressor variables had VIF higher than 5 in fully adjusted models, indicating that overlapping information could diminish independent effects of each factor on probable PTSD (39, 40). Also, we calculated the events per variable (EPV) (41) to ensure the stability of two fully adjusted models. Although both EPVs fell within the range of 5–9 (duty type model: 75/12 = 6.25, stressor model: 75/15 = 5), the ratio for acceptable estimates (42), they fell below the traditional rule of EPV ≥ 10 (41) which indicates potential overfitting.

Therefore, we further conducted logistic regression analyses controlling for only baseline characteristics and included each duty type or stressor to eliminate the overlapping influence of similar duty types or stressors and a potential overfitting (partially adjusted models). Each partially adjusted duty type/stressor model had 6 predictors (*5 baseline + 1 duty type/stressor for each model*) with 75 probable PTSD cases and 660 FIs without probable PTSD (*N* = 735). The VIFs for variables included in all partially adjusted models were found to be < 5, and the EPVs for all partially adjusted models were found to be ≥10 (75/6 = 12.5), all of which satisfy the traditionally recommended guidelines for logistic regression. All statistical analyses were conducted using IBM SPSS Statistics (Version 29).

## 3 Results

### 3.1 Demographic information

A total of 735 FIs responded to the survey, and there was no missing data. Their basic characteristics are presented in Table 1.

**Table 1:** Sample characteristics (*N* = 735).

Characteristics	*N* (%)
Gender	
Male	536 (72.9%)
Female	199 (27.1%)
Age	
20–29	26 (3.5%)
30–39	305 (41.5%)
40–49	242 (32.9%)
50+	162 (22.0%)
Household characteristics	
Unmarried/Divorced/Bereaved/etc.	171 (23.3%)
Married	564 (76.7%)
Having a child	489 (66.5%)
Engagement type	
Directly deployed in Jeju Air crash scene	204 (27.8%)
Indirectly deployed in Jeju Air crash scene	20 (2.7%)
Total deployed in Jeju Air crash scene (engaged FIs)	224 (30.5%)
Did not participate in Jeju Air crash recovery (non-deployed FIs)	511 (69.5%)
Entry route	
Open recruitment for entry-level police officers / Competitive recruitment for police administration	467 (63.5%)
Competitive recruitment for forensic science positions	244 (33.2%)
Others (e.g., foreign affairs, victim support officers, police officer cadet, police academy graduates)	24 (3.3%)
Job type	
Field-based police officers	536 (72.9%)
Medicolegal death investigators	131 (17.8%)
Others (e.g., Administrator, crime analysis, forensic psychologist, etc.)	68 (9.3%)
Rank	
*Field-based police officers*	
Police officer (Soongyeong)	44 (6.0%)
Senior Policeman (Gyeongjang)	82 (11.2%)
Sergeant (Gyeongsa)	181 (24.6%)
Inspector (Gyeongwi)	215 (29.3%)
Senior inspector (Gyeonggam)	71 (9.7%)
Superintendent (Gyeongjeong)	11 (1.5%)
*Medicolegal death investigators*	
Grade 9	34 (4.6%)
Grade 8	40 (5.4%)
Grade 7	37 (5.0%)
Grade 6	14 (1.9%)
Grade 5	5 (0.7%)
Unspecified	1 (0.1%)
Years of service	
≤5 years	164 (22.3%)
6–10 years	188 (25.6%)
11–15 years	101 (13.7%)
16–20 years	127 (17.3%)
≥21 years	155 (21.1%)
Years of service (forensic investigation)	
≤5 years	357 (48.6%)
6–10 years	176 (23.9%)
11–15 years	96 (13.1%)
16–20 years	77 (10.5%)
≥21 years	29 (3.9%)
Exposure to severely damaged human remains	
Less than 5 cases	263 (35.8%)
≥5 cases	472 (64.2%)
Exposure to a massive number of human remains	
Less than 5 cases	659 (89.7%)
≥5 cases	76 (10.3%)

About one in three (30.5%, *n* = 224) were engaged in response to Jeju Air crash (engaged FI). FIs who were directly engaged in recovery at Muan Airport after the incident accounted for 27.8% of total respondents (directly engaged FI, *n* = 204). FIs who were involved in duties related to the recovery of MFI but not directly deployed to the scene accounted for 2.7% of total respondents (indirectly engaged FI, *n* = 20). This revealed that most of the engaged FIs were directly deployed to the disaster response. The mean years of service as a police officer was 16.4 years (SD = 8.87, median = 11 years), while mean length of service as an FI was 7.97 (SD = 6.28, median = 6 years). Most respondents (72.5%, *n* = 533) had less than 10 years of experience as an FI.

### 3.2 Overall mental health outcomes and subgroup comparisons (engaged vs. non-deployed)

Table 2 presents the overall mental health status of FIs and prevalence comparisons between engaged and non-deployed FIs. For investigating mental health and influencing key factors among engaged FIs, the indirectly and directly engaged FIs (*n* = 20 and *n* = 204, respectively) were combined (engaged FI; *n* = 224). To justify the consolidation, we conducted sensitivity analyses by comparing fully adjusted logistic regression models (covariates: basic characteristics, MFI stressors or duty types) of engaged FIs (*n* = 224) and directly engaged FIs (*n* = 204). Two models showed similar results, especially both of which showed the same significant variable in MFI stressor model [emotional identification with the bereaved; engaged FIs OR = 3.29 (1.14, 9.48); directly engaged FIs OR = 3.78 (1.22, 11.69)]. Detailed results are presented in Supplementary Tables 1, 2.

**Table 2:** Mental health prevalence of overall, engaged and non-deployed forensic investigators (FIs).

Measure	Classification	Total FIs (*N* = 735)		Engaged FIs (*n* = 224)		Non-deployed FIs (*n* = 511)		Engaged vs. Non-deployed (prevalence of probable symptoms)	
		M (SD)	*N* (%) [95% CI]	M (SD)	*N* (%) [95% CI]	M (SD)	*N* (%) [95% CI]	Chi-square	Effect size (Cramer’s *V*)
IES-R	Reported traumatic event	11.29 (14.37)	368 (50.1)	13.90 (16.27)	148 (66.1)	10.15 (13.31)	220 (43.1)	$x^{2}=8.7$ df=1 p=.003	0.109
	IES-R ≥ 25		92 (12.5) [10.1, 14.9]		36 (16.1) [11.3, 20.9]		56 (11.0) [8.3, 13.7]		
	*Probable PTSD (≥25 & reported traumatic event)*		*75 (10.2) [8.0, 12.4]*		*34 (15.2) [10.5, 19.9]*		*41 (8.0) [5.7, 10.4]*		
PHQ-9	No depression (0–4)	3.43 (4.25)	527 (71.7)	3.69 (4.61)	154 (68.8)	3.31 (4.08)	373 (73.0)	$x^{2}=0.34$ df=1 p=.559	0.022
	Mild depression (5–9)		149 (20.3)		54 (24.1)		95 (18.6)		
	Moderate depression (10–19)		52 (7.1)		12 (5.4)		40 (7.8)		
	Severe depression (≥20)		7 (1.0)		4 (1.8)		3 (0.6)		
	*Probable depression (≥10)*		*59 (8.0) [6.1, 10.0]*		*16 (7.1) [3.8, 10.5]*		*43 (8.4) [6.0, 10.8]*		
GAD-7	Not anxious (0–4)	1.88 (3.13)	608 (82.7)	1.96 (3.09)	179 (79.9)	1.84 (3.15)	429 (84.0)	$x^{2}=0.86$ df=1 p=.355	0.034
	Mild anxiety (5–9)		104 (14.1)		40 (17.9)		64 (12.5)		
	Moderate anxiety (10–14)		15 (2.0)		3 (1.3)		12 (2.3)		
	Severe anxiety (≥15)		8 (1.1)		2 (0.9)		6 (1.2)		
	*Probable anxiety (≥10)*		*23 (3.1) [1.9, 4.4]*		*5 (2.2) [0.3, 4.2]*		*18 (3.5) [1.9, 5.1]*		
MHS: S	No suicidality (0)	0.34 (1.31)	649 (88.3)	0.32 (1.18)	199 (88.8)	0.34 (1.36)	450 (88.1)	$x^{2}=0.00$ df=1 p=.995	0.000
	Mild suicidality (1)		40 (5.4)		11 (4.9)		29 (5.7)		
	Moderate suicidality (2)		9 (1.2)		1 (0.4)		8 (1.6)		
	Severe suicidality (≥3)		37 (5.0)		13 (5.8)		24 (4.7)		
	*Probable suicidality*		*46 (6.3) [4.5, 8.0]*		*14 (6.3) [3.1, 9.4]*		*32 (6.3) [4.2, 8.4]*		
AUDIT-C	Currently consuming alcohol	3.72 (2.73)	619 (84.2)	3.85 (2.84)	189 (84.4)	3.66 (2.68)	430 (84.1)	$x^{2}=2.88$ df=1 p=.090	0.063
	*Probable heavy alcohol use (≥8)*		*83 (11.3) [9.0, 13.6]*		*32 (14.3) [9.7, 18.9]*		*51 (10.0) [7.4, 12.6]*		
Total	Experiencing one or more probable symptoms (depression, anxiety, suicidality, alcohol use, PTSD)	145 (19.7) [16.9, 22.6]		50 (22.3) [16.9, 27.8]		95 (18.6) [15.2, 22.0]		$x^{2}=1.37$ df=1 p=.242	0.043

According to the self-reported experiences of traumatic events, about half (*n* = 368, 50.1%) nominated their most distressing event at their work. There were a few FIs who nominated two or more experiences (*n* = 12, 1.6%) at once. Among traumatic events, there were routine events such as non-MFI accidents (e.g., traffic, industrial) (*n* = 41, 11.1%), homicides (*n* = 54, 14.7%), suicides (*n* = 25, 6.8%), and other unspecified events (*n* = 115, 31.3%). Some FIs nominated MFIs such as Sewol ferry sinking (*n* = 14, 3.8%), Itaewon crowd crush (*n* = 32, 8.7%), and the recent Jeju Air crash (*n* = 74, 20.1%), and other MFIs (*n* = 26, 7.1%). Among engaged FIs, two in three (*n* = 148, 66.1%) nominated their most distressing event at their work, half of whom nominated the Jeju Air crash (*n* = 74, 33.0%). The proportion of FIs who reported the most traumatic event among engaged FIs was significantly higher than that of non-deployed FIs ( $x^{2}=33.0,\mathrm{df}=1,p<.001)$ , and its effect size is small ( ${\text{Cramer}}^{'}s\;V=.212)$ . Post-hoc analysis using adjusted standardized residuals revealed that the engaged group was significantly more likely to report the most traumatic event than expected ( z=5.75,p<.01) , while non-deployed group was significantly less likely ( z=−5.75,p<.01) .

The prevalence of probable PTSD among overall FIs was 10.2% [95% CI: (8.0, 12.4), *n* = 75]. Among the FIs who were directly involved in MFI response, almost one in six [15.2, 95% CI: (10.5, 19.9), *n* = 34] reported probable PTSD symptoms. This probable PTSD prevalence of engaged FIs was significantly higher than that of non-deployed FIs ( $x^{2}=8.7,\mathrm{df}=1,p=.003)$ and its effect size is small ( ${\text{Cramer}}^{'}s\;V=.109)$ . Post-hoc analysis using adjusted standardized residuals revealed that the engaged group was significantly more likely to have probable PTSD than expected ( z=2.95,p<.01) , while non-deployed group was significantly less likely ( z=−2.95,p<.01) .

The prevalence and group differences of general mental health disorders among FIs were also assessed. The overall prevalence of probable clinical depression (moderate and severe) was 8.0% [95% CI: (6.1, 10.0), *n* = 59], and the prevalence of engaged FIs was not significantly different from that of non-deployed FIs ( $x^{2}=0.34,\mathrm{df}=1,p=.559).$ The overall proportion of probable clinical anxiety (moderate and severe) was 3.1% [95% CI: (1.9, 4.4), *n* = 23], and the prevalence of engaged FIs was not significantly different from that of non-deployed FIs ( $x^{2}=0.86,\mathrm{df}=1,p=.355)$ . The proportion of the respondents who reported probable clinical levels of suicide risk (moderate and high risk) was 6.3% [95% CI: (4.5, 8.0), *n* = 46], and the prevalence of engaged FIs was not significantly different from that of non-deployed FIs ( $x^{2}=0.00,\mathrm{df}=1,p=.995)$ . The proportion of heavy alcohol consumption was 11.3% [95% CI: (9.0, 13.6), *n* = 83]. The alcohol use prevalence of engaged FIs was higher than that of non-deployed FIs, marginally but not significantly different ( $x^{2}=2.88,\mathrm{df}=1,p=.090)$ .

Overall, 19.7% [95% CI: (16.9, 22.6), *n* = 145] of FIs reported that they are experiencing more than one probable mental health disorder (probable PTSD, depression, anxiety, suicidality, alcohol use). The overall mental health disorder prevalence of engaged FIs [22.3, 95% CI: (16.9, 27.8), *n* = 50] was higher than that of non-deployed FIs [18.6, 95% CI: (15.2, 22.0), *n* = 95], but not significantly different ( $x^{2}=1.37,\mathrm{df}=1,p=.242)$ .

### 3.3 Key factors associated with probable PTSD symptoms of engaged FIs

#### 3.3.1 Duty types

**Figure 1:**
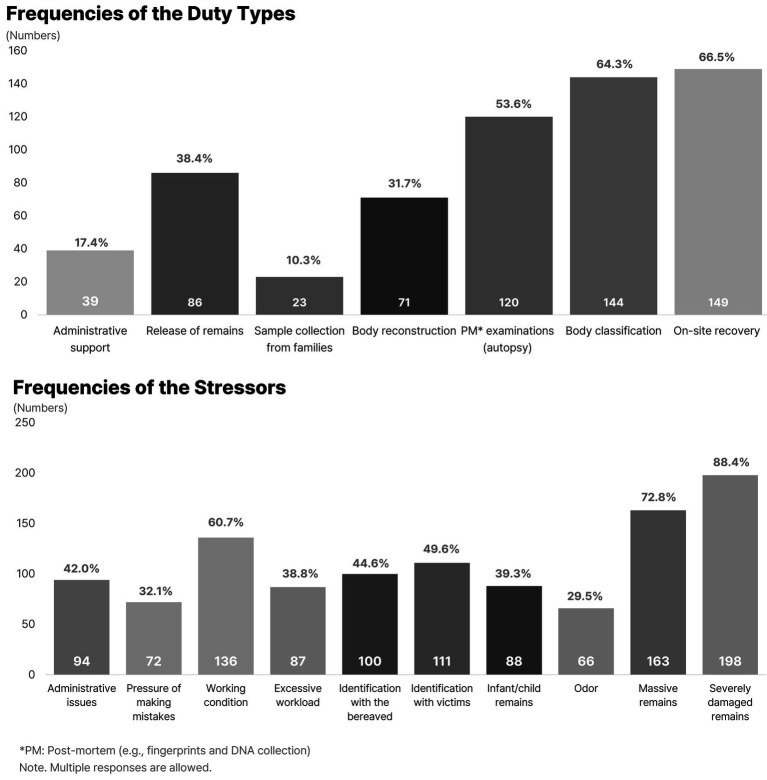
Frequencies of the MFI duty types and stressors (*n* = 224).

The duty types assigned to engaged FIs (*n* = 224) involved in the response were as follows. About two in three engaged FIs (66.5%, *n* = 149) were engaged in the recovery (e.g., searching for evidence and human remains). In addition, 64.3% (*n* = 144) participated in body classification, 53.6% (*n* = 120) in autopsy (e.g., fingerprint and DNA collection), 31.7% (*n* = 71) in body reconstruction, and 10.3% (*n* = 23) in collection of samples from bereaved family members. Additionally, 38.4% (*n* = 86) of the respondents were assigned to the release of identified remains to families, which was not a routine part of DVI procedures. For administrative support, 17.4% (*n* = 39) of the FIs were involved. The frequencies of each duty type experienced by FIs involved in the response are shown in Figure 1.

The mean score of subjective psychological distress for recovery was 2.09 (SD = 0.86). The mean distress level was 2.16 (SD = 0.86) for body classification, 2.06 (SD = 0.91) for post-mortem examinations, and 2.38 (SD = 0.82) for body reconstruction. The mean score of subjective distress was the highest for release of identified remains to families (M = 2.62, SD = 1.00). The mean distress level of respondents was 2.21 (SD = 0.90) for sample collection from families, 1.92 (SD = 0.81) for administrative support. Except for administrative support, mean distress levels of all duty types scored over 2 (somewhat distressed). The psychological distress levels of each duty type experienced by FIs involved in the response are shown in Figure 1.

#### 3.3.2 Stressors

Most of the FIs involved were distressed by severely damaged human remains (88.4%, *n* = 198) and a massive number of human remains (72.8%, *n* = 163). They reported the experience of distressing odor (29.5%, *n* = 66), infant/child remains (39.3%, *n* = 88), identification with the victims (49.6%, *n* = 111), and identification with the bereaved while interacting with the family members of the victims (44.6%, *n* = 100). For the working environment, FIs were distressed by the excessive workload (38.8%, *n* = 87), poor working conditions (60.7%, *n* = 136), pressure of making mistakes (32.1%, *n* = 72), and the distress related to administrative systems (42.0%, *n* = 94). Other various kinds of stressors were reported, including pressure from the unorganized situation, physical and mental fatigue, and being filmed by the media. The frequencies of each stressful event experienced by FIs involved in the response are shown in Figure 1.

**Figure 2:**
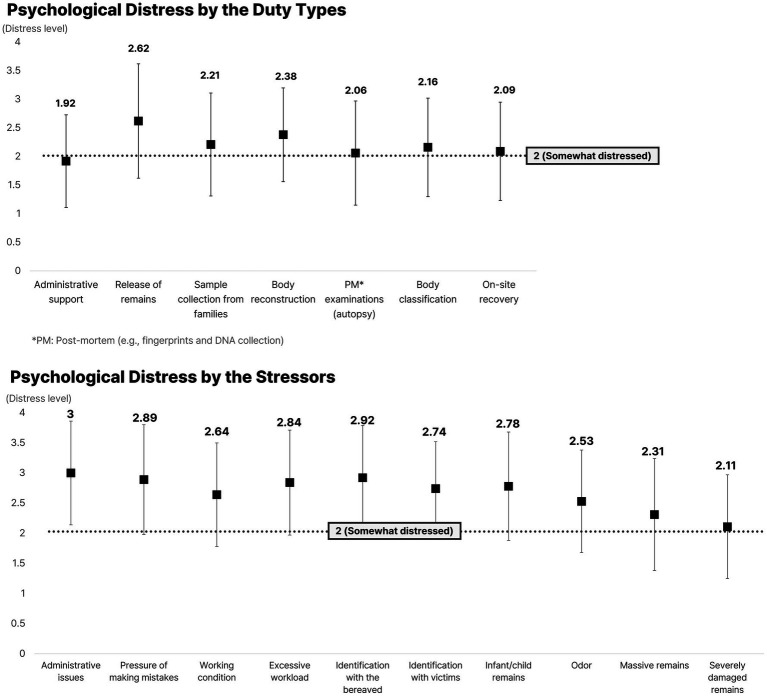
Means and standardized deviations of psychological distress of the MFI duty types and stressors (*n* = 224).

The mean distress level for exposure to severely damaged human remains was 2.11 (SD = 0.86). Mean distress level was 2.31 (SD = 0.93) for massive numbers of remains, 2.53 (SD = 0.85) for distressing odors, and 2.78 (SD = 0.90) for infant/child remains. For identification with victims, the mean distress score was 2.74 (SD = 0.78), and if they identified emotionally with the family members of the victims, the mean distress score was even higher (M = 2.92, SD = 0.87). The mean score of distress was 2.84 (SD = 0.87) for excessive workload, 2.64 (SD = 0.86) for the poor working conditions, 2.89 (SD = 0.91) for pressure from the potential errors, and 3.00 (SD = 0.86) for administrative issues. The psychological distress levels of each stressful event experienced by FIs involved in the response are shown in Figure 2.

Most of the engaged FIs involved in the incident experienced more than one stressful event in the scene, and the mean of psychological distress levels due to all types of stressors scored above 2 (somewhat distressed). Among the events, mean distress scores resulted from exposure to infant/child remains, contact with the family members, excessive workload, pressure from making mistakes, and administrative issues almost reached 3 (distressed) in average.

#### 3.3.3 Duty types and stressors associated with probable PTSD

According to the results of correlation tests with Bonferroni correction, body reconstruction, exposure to infant or child remains, emotional identification with the victim and the bereaved, excessive workload, poor working conditions, and pressure of making mistakes were significantly associated with one or more PTSD symptoms: avoidance, hyperarousal, intrusion, and numbing among the duty types. The overall correlation matrix between PTSD symptoms and duty types/stressors in response of the crash is as below (Table 3).

**Table 3:** The associations between PTSD symptoms and duty types/stressors in response to Jeju Air crash (*n* = 224).

Duty types	IES-R scores				
	Avoidance	Hyperarousal	Intrusion	Numbing	Total
On-site recovery	0.048	0.058	0.031	0.021	−0.035
Body classification	0.114	0.175	0.164	0.108	0.144
Autopsy	0.131	0.141	0.160	0.126	0.119
Body reconstruction	0.254^*^	0.265^*^	0.250^*^	0.239^*^	0.263^*^
Collection of samples from bereaved family members	0.223	0.173	0.172	0.193	0.215
Release of identified remains to families	0.224	0.217	0.173	0.177	0.131
Administrative support	0.104	0.152	0.127	0.081	0.223
Stressors					
Exposure to (severely) damaged human remains	0.028	−0.025	0.068	−0.024	0.017
Exposure to a massive number of human remains	0.074	0.147	0.133	0.153	0.119
Exposure to infant or child remains	0.185	0.248^*^	0.186	0.125	0.204
(Distressing) odors	0.111	0.101	0.156	0.137	0.140
Emotional identification with the victim	0.330^*^	0.222	0.326^*^	0.186^*^	0.323^*^
Emotional identification with the bereaved	0.354^*^	0.370^*^	0.333^*^	0.285^*^	0.371^*^
Excessive workload	0.196	0.272^*^	0.253^*^	0.240^*^	0.251^*^
Poor working conditions	0.190	0.232^*^	0.245^*^	0.282^*^	0.256^*^
Pressure of making mistakes	0.226	0.270^*^	0.281^*^	0.254^*^	0.277^*^
Unorganized system and other administrative system-related stressors	0.097	0.210	0.108	0.154	0.119

Spearman’s rank correlation analysis.

^*^p < 0.05 (Bonferroni corrected).

The logistic regression analyses with experiences of duty types controlling for confounders were conducted (Table 4). According to the results of partially adjusted models controlling for baseline characteristics, body classification [OR = 2.13; 95% CI: (1.25, 3.65)], autopsy [OR = 1.91; 95% CI: (1.09, 3.34)], body reconstruction [OR = 2.73; 95% CI: (1.47, 5.09)], collection of samples from bereaved family members [OR = 3.71; 95% CI: (1.40, 9.84)], and administrative support [OR = 2.32; 95% CI: (1.02, 5.30)] were significantly associated with probable PTSD among FIs. In the fully adjusted model controlling for baseline characteristics and all duty types, there were no duty types significantly associated with probable PTSD among FIs.

**Table 4:** Logistic analysis of associated MFI duty types with probable PTSD (*N* = 735).

Predictors		Probable PTSD [Odds Ratio (95% CI)]	
		Partially adjusted^a^	Fully adjusted^b^
		*n* = 735	*n* = 735
Step 1. Baseline characteristics	Sex	0.80 [0.43, 1.48]^c^	0.83 [0.44, 1.56]
	Age	1.03 [0.99, 1.08]^c^	1.04 [0.99, 1.08]
	Service year	1.01 [0.96, 1.07]^c^	1.02 [0.96, 1.07]
	5+ exposures to severely damaged bodies	4.97 [2.21, 11.19]^***,^^c^	4.27 [1.87, 9.77]^***^
	5+ exposures to massive damaged bodies	1.48 [0.76, 2.87]^c^	1.32 [0.65, 2.65]
Step 2. MFI duty types	On-site recovery	1.24 [0.70, 2.18]	0.57 [0.25, 1.29]
	Body classification	2.13 [1.25, 3.65]^**^	2.27 [0.89, 5.83]
	Autopsy	1.91 [1.09, 3.34]^*^	0.91 [0.37, 2.27]
	Body reconstruction	2.73 [1.47, 5.09]^**^	2.12 [0.85, 5.25]
	Collection of samples from bereaved family members	3.71 [1.40, 9.84]^**^	2.15 [0.69, 6.75]
	Release of identified remains to families	1.53 [0.81, 2.92]	0.70 [0.28, 1.74]
	Administrative support	2.32 [1.02, 5.30]^*^	1.41 [0.53, 3.72]
*R* ^2^	0.153		
*x*^2^/df	56.3/12^***^		

^*^p < 0.05, ^**^p < 0.01, ^***^p < 0.001.

a Covariates: baseline characteristics and each duty type (6 predictors), number of events: 75 (potential PTSD).

b Covariates: baseline characteristics, all duty types (12 predictors), number of events: 75 (potential PTSD).

c Results of the analysis including only baseline characteristics.

The logistic regression analyses with experiences of stressors in Muan were also conducted (Table 5). According to the results of partially adjusted models, exposure to a massive number of human remains [OR = 1.89; 95% CI: (1.12, 3.21)], exposure to infant or child remains [OR = 2.08; 95% CI: (1.12, 3.84)], odors [OR = 2.10; 95% CI: (1.07, 4.12)], emotional identification with the victim [OR = 2.12; 95% CI: (1.21, 3.74)], emotional identification with the bereaved [OR = 3.32; 95% CI: (1.89, 5.82)], excessive workload [OR = 2.40; 95% CI: (1.32, 4.34)], poor working conditions [OR = 2.01; 95% CI: (1.17, 3.44)], pressure of making mistakes [OR = 3.29; 95% CI: (1.77, 6.12)], and unorganized system and other administrative system-related stressors [OR = 1.86; 95% CI: (1.03, 3.35)] were significantly associated with probable PTSD among FIs. In the fully adjusted model, emotional identification with the bereaved was significantly associated with probable PTSD [OR = 3.40; 95% CI: (1.22, 9.44)] among FIs.

**Table 5:** Logistic analysis of associated MFI stressors with probable PTSD (*N* = 735).

Predictors		Probable PTSD [Odds Ratio (95% CI)]	
		Partially adjusted^a^	Fully adjusted^b^
		*n* = 735	*n* = 735
		75 events (Probable PTSD)	75 events (Probable PTSD)
		6 predictors	15 predictors
Step 1. Baseline characteristics	Sex	0.80 [0.43, 1.48]^c^	0.93 [0.48, 1.78]
	Age	1.03 [0.99, 1.08]^c^	1.03 [0.98, 1.08]
	Service year	1.01 [0.96, 1.07]^c^	1.02 [0.96, 1.08]
	5+ exposures to severely damaged bodies	4.97 [2.21, 11.19]^***,^^c^	4.54 [1.97, 10.47]^***^
	5+ exposures to massive damaged bodies	1.48 [0.76, 2.87]^c^	1.12 [0.55, 2.30]
Step 2. MFI stressors	Exposure to (severely) damaged human remains	1.58 [0.94, 2.65]	0.40 [0.10, 1.51]
	Exposure to a massive number of human remains	1.89 [1.12, 3.21]^*^	0.97 [0.25, 3.74]
	Exposure to infant or child remains	2.08 [1.12, 3.84]^*^	1.24 [0.50, 3.04]
	(Distressing) odors	2.10 [1.07, 4.12]^*^	1.19 [0.48, 2.98]
	Emotional identification with the victim	2.12 [1.21, 3.74]^**^	1.19 [0.48, 2.91]
	Emotional identification with the bereaved	3.32 [1.89, 5.82]^***^	3.40 [1.22, 9.44]^*^
	Excessive workload	2.40 [1.32, 4.34]^**^	1.14 [0.42, 3.08]
	Poor working conditions	2.01 [1.17, 3.44]^*^	1.09 [0.34, 3.51]
	pressure of making mistakes	3.29 [1.77, 6.12]^***^	1.98 [0.77, 5.08]
	Unorganized system and other administrative system-related stressors	1.86 [1.03, 3.35]^*^	0.92 [0.37, 2.28]
*R* ^2^	0.168		
*x*^2^/df	62.1/15^***^		

^*^p < 0.05, ^**^p < 0.01, ^***^p < 0.001.

a Covariates: baseline characteristics and each duty type.

b Covariates: baseline characteristics, all duty types.

c Results of the analysis including only baseline characteristics.

## 4 Discussion

### 4.1 Prevalence of probable PTSD and comorbid conditions of overall FIs

The present study provides compelling evidence of elevated probable PTSD rates and comorbid mental health challenges among FIs in Korea. Our findings revealed that approximately one in ten FIs [10.2, 95% CI: (8.0, 12.4)] screened positive for probable PTSD, suggesting that a substantial proportion of these professionals experience significant psychological distress. Among those who identified a traumatic event, nearly 60% cited routine occupational incidents—such as homicides and accidents—rather than major disasters. This aligns with previous meta-analytical evidence suggesting that FRs routinely exposed to trauma, such as firefighters, may exhibit higher PTSD prevalence than those responding to a single catastrophic event (43). Thus, the cumulative nature of routine traumatic exposure appears to be critically associated with probable PTSD, independent of the Jeju Air crash.

Furthermore, the elevated mental health risk of FIs extends beyond PTSD. Nearly 20% of the sample [19.7, 95% CI: (16.9, 22.6)] met the criteria for at least one significant mental health condition, including depression, anxiety, heavy alcohol use, and high suicide risk. Interestingly, the prevalence of depression (8.0%) and anxiety (3.1%) was considerably lower than the ~20% reported among FIs in the UK (44). This discrepancy likely reflects a stigmatizing organizational culture that discourages FIs from disclosing general mental health issues. While PTSD among Korean FIs has gained recent public and academic attention, conditions like depression and substance use remain largely under-addressed. The culture of “enduring in silence” among Korean police investigators, triggered by fear of vulnerability and feelings of isolation (45), could affect the underreporting of other mental health disorders. As the first study to explore a broad spectrum of mental health indicators in this population, our findings underscore the need for comprehensive screening and longitudinal monitoring of FIs’ overall mental well-being.

### 4.2 Association of the Jeju Air crash and duty-related factors associated with probable PTSD

A significantly higher percentage of engaged FIs reported that they had experienced traumatic events during their FI duties. The prevalence of probable PTSD among engaged FIs [15.2, 95% CI: (10.5, 19.9)] was significantly higher than that of the non-deployed, which is consistent with previous findings estimating PTSD prevalence for FRs after MFIs (10.2–16.7%) (6–8). Even though more than half of engaged FIs reported the most traumatic event other than Jeju Air crash, the cumulative characteristics of traumatic events among routine responders (43, 46) could explain the association of the recent MFI with higher PTSD vulnerability. This finding confirms that a single involvement of MFI is significantly associated with probable PTSD for FIs. Considering the small but significant effect of MFI engagement on FIs (Cramer’s *V* = 0.109), which could be a potential burden across the FIs, timely mental health screening and psychological support should be conducted for FIs who both are directly and indirectly deployed to MFI sites.

Among tasks and potential stressors, emotional identification with the bereaved emerged as the sole robust factor of probable PTSD in the fully adjusted logistic model [OR = 3.40, 95% CI (1.22, 9.44)]. Furthermore, emotional identification with victims and the bereaved both were significantly associated with traumatic symptoms and probable PTSD according to the correlation and exploratory partial logistic analyses. This indicates that the emotional identification-related experience is one of the most crucial stressors related to probable PTSD. Due to logistical confusion on site, about half of the engaged FIs in Muan unexpectedly performed the unaccustomed duty of releasing remains to bereaved families, and it forced FIs into an emotional confrontation with the intense anger and grief of the bereaved who suddenly had lost their beloved ones. This experience appeared to be linked to emotional identification, previously known to frequently occur among FIs deployed in MFIs (16, 47). The present study showed that the novel experience of emotional confrontation in MFI sites was related to intense feelings of identification, acting as unique MFI site-specific factors associated with probable PTSD among FIs. The underlying mechanism of this phenomenon should be further studied, since emotional identification is likely to be worsened by subsequent negative psychological vulnerabilities such as compassion fatigue, moral injury, and burnout (17).

Furthermore, exploratory partial logistic regression and correlation analyses suggested that handling damaged remains repetitively and continuously was significantly related to probable PTSD. The majority of FIs were involved in handling severely fragmented and thermally damaged remains during DVI procedures, and this distressing experience appears to trigger probable PTSD. While FIs were found to be distressed by the sheer exposure to human remains, this did not result in a significant association with PTSD symptoms. However, continuous exposure to sensory details during DVI, such as massive remains, infant or child remains, and distressing odors, appeared to significantly exacerbate further traumatic distress and PTSD symptoms. The study stresses that the DVI procedure entails detailed and continuous exposure to the traumatic scenes, which makes engaged FI one of the high-risk FRs of MFIs.

Our findings also suggest that non-traditional occupational stressors can be psychologically damaging as traumatic exposure itself. Aligned with previous findings (19, 48), duty-related stressors such as excessive workload, poor working conditions, and pressure of making mistakes were all associated with PTSD symptoms according to correlation analysis. Partially adjusted logistic regression models conducted as exploratory also indicated that the duty-related stressors were significantly associated with probable PTSD. This underscores the need for authorities to move beyond “trauma-related” interventions and address structural stressors—such as overwork, resource shortages, and pressure of making mistakes—while considering the individual mental health prevention and intervention of engaged FIs.

These three factors (emotional identification, DVI procedure handling damaged remains, and work-related stressors) are all associated with the aforementioned main factors linked to probable PTSD at MFI sites: unpredictability, uncontrollability, and threat (16). In other words, FIs dispatched to Muan appeared to be unexpectedly exposed to catastrophic scenes and the intense emotions of the bereaved without any notification. They were psychologically threatened by continuously handling damaged bodies during DVI and experiencing intense emotional identification with the victims. However, challenging working environments prevented FIs from managing these stressors with a sense of control. These stressors accumulated and appeared to be associated with a higher prevalence of probable PTSD and other mental health conditions among engaged FIs.

### 4.3 Strengths, limitations, and implications

The present study possesses a number of strengths. This study included a nationwide survey sample of FIs in Korea. We also used validated psychological assessment instruments, detailed questionnaires on the most traumatic event during duty, and specific duty types and stressors experienced at MFI sites. Notably, few studies have empirically explored the mental health status of FIs in Korea following an MFI. The study succeeded in specifically elucidating which FI duty types and stressors were stressful and associated with higher PTSD vulnerability, including catastrophic exposure, identification-related experience and poor workplace condition.

This study yet holds several limitations. The reliance on self-report measures and convenient sampling which can be subject to bias within the culture of FIs stigmatizing mental health, could result in underreport of mental health symptoms. This stigmatizing culture among the uniformed personnel appears to affect a relatively lower rate of complete survey response (66.8%). In addition, the cross-sectional design limits our ability to track the longitudinal phase of the mental health condition during and after MFI response. Although the time between exposure (December 2024) and outcome assessment (September 2025) was approximately 9 months, the single-timepoint design precludes causal inference. The associations reported here should be interpreted as cross-sectional correlates of probable PTSD rather than established risk factors. This caution is particularly relevant, as current PTSD symptom severity may influence the reporting of stressor types and intensities through mood-congruent memory effects (49). Also, the study used the presence of the most distressing event while working as a criterion for probable PTSD in addition to the IES-R cutoff score to measure the prevalence of probable PTSD. However, this approach has the limitation of potentially excluding participants who experience symptoms but avoid clearly identifying the specific event due to traumatic symptoms. In future research, further methods such as additional questions could be considered. Lastly, as the study focused primarily on exterior environmental factors such as duty types, subsequent studies could further explore the internal mechanisms of identification with victims, including moral injury (50), compassion fatigue (51), or cognitive-emotional strategies (52) that may affect the duty-related psychological symptoms.

The present study has significant implications for mental health of FIs in Korea. We documented that FIs were routinely exposed to traumatic events, therefore highly vulnerable to probable PTSD and mental health conditions. This necessitates a focus on prevention and intervention measures. The Korean National Police Agency implemented steps with psychotherapy service initiatives for police officers including FIs, Mind Accompanying Center (*Maeum Donghaeng Center*). The centers show high utilization rate, possibly due to their universal installation nationwide and the mandatory counseling policy. However, according to the recent report, FIs reported that the current service appeared to be generic rather than specialized for their psychological issues, compulsory rather than voluntary, preventing them from receiving the specific service when and what they need (26).

Policy initiatives should ensure that FIs receive appropriate psychological treatment specialized for PTSD and major mental health issues. This could be implemented based on the principles of evidence-based practice in psychology, consisting of clinical expertise (e.g., M.A.+ in the field of mental health, 1+ years of training in psychotherapy, expertise in traumatic exposure), best available research evidence [e.g., trauma-focused cognitive behavioral therapy (TF-CBT), prolonged exposure (PE)], and patient characteristics, culture, and preferences (therapist sensitivity regarding FI experience and trauma) (53, 54). We recommend implementing a stepped-care model (55) based on symptom severity. For example, for relatively low-risk groups, low-intensity support such as peer support, and team recovery programs could be provided. For the high-risk groups, high-intensity support such as TF-CBT by psychotherapists is recommended. Regular mental health monitoring, especially after the large-scale MFI response, can help categorize FIs and provide therapeutic intervention to specialized services in a timely manner.

Systematic measures should also be implemented to address related factors prevalent at MFI sites. Authorities must promote predictability, controllability, and coping strategies against MFIs to mitigate potential threats on site. For example, strengthening pre-deployment briefings including key information (e.g., the scale of the incident, the on-site situation, and their roles and tasks) can reduce their surprise and confusion. Basic recovery resources such as mobile rest areas should also be provided to alleviate work overload at disaster sites. Also, training protocols for potential FIs on MFI sites should include programs based on evidence-based treatments such as affective modulation and stress management in TF-CBT to prevent traumatic experiences due to excessive emotional identification and occupational stress in advance (56), which are potential site-specific factors of probable PTSD according to the study.

Ultimately, we recommend that forensic organizations adopt the principles of Trauma-Informed Care (TIC) (57). This paradigm shift entails fostering a workplace culture that acknowledges the universality of trauma—recognizing that any investigator is susceptible to PTSD and that seeking psychological support is a professional strength rather than an occupational failure. By establishing evidence-based, trauma-informed initiatives, organizations can safeguard their personnel and reinforce their vital mission, thereby facilitating the pursuit of truth in the aftermath of disasters.

## 5 Conclusion

The present study investigated the nationwide psychological health of Korean FIs while specifically examining the association between the duty-related experience of Jeju Air crash and mental health. Engaged FIs had a significantly higher risk of probable PTSD, which was significantly associated with DVI procedure on MFI scenes, emotional identification, and challenging working conditions. As this is the first study to explore the mental health of FIs after an MFI, the findings emphasize the importance of mental health screening and psychological services for FIs deployed to disaster sites. Ultimately, it is imperative to provide PTSD-specific evidence-based psychotherapy to FIs, and to be systematically empowered based on the principles of TIC.

## Data Availability

The raw data supporting the conclusions of this article will be made available by the authors, without undue reservation.
