# Supplementary material for: The psychological health of forensic investigators following mass fatality incidents: a cross-sectional study 9 months after the Jeju Air crash in South Korea
Source: Front Public Health. 2026 May 29;14:1819655. doi: 10.3389/fpubh.2026.1819655 (PMC13259849; doi:10.3389/fpubh.2026.1819655)
Supplement: Supplementary file 1 [file Data_sheet_1.docx]

STROBE Statement—Checklist of items that should be included in reports of cross-sectional studies

|  | Item No | Recommendation | Relevant text from manuscript |
| --- | --- | --- | --- |
| **Title and abstract** | 1 | (*a*) Indicate the study’s design with a commonly used term in the title or the abstract | (Title) ‘The Psychological Health of Forensic Investigators following Mass Fatality Incidents: A Cross-sectional Study Nine Months after the Jeju Air Crash in South Korea’ |
|  |  | (*b*) Provide in the abstract an informative and balanced summary of what was done and what was found | (Abstract) Therefore, the current cross-sectional survey was conducted among a total of 735 FIs, including 224 individuals who had been engaged in the Jeju Air crash site nine months prior to the study. Mental health outcomes, including probable PTSD (IES-R-K defined), depression (PHQ-9 defined), anxiety (GAD-7 defined), alcohol use (AUDIT-C defined), and suicide risk (MHS:S defined), were evaluated. |
| Introduction | | |  |
| Background/rationale | 2 | Explain the scientific background and rationale for the investigation being reported | (Introduction) FRs are constantly exposed to psychologically traumatic events such as serious injuries or deaths and being distressed by strenuous working conditions, all of which are significant risk factors of post-traumatic stress disorder (PTSD) |
| Objectives | 3 | State specific objectives, including any prespecified hypotheses | (Introduction) The primary objective of this study is to empirically evaluate the mental health status of overall FIs and FIs who participated in on-site recovery of Jeju Air crash nine months after the incident. |
| Methods | | |  |
| Study design | 4 | Present key elements of study design early in the paper | (Abstract) Therefore, the current cross-sectional survey was conducted among a total of 735 FIs, including 224 individuals who had been engaged in the Jeju Air crash site nine months prior to the study. |
| Setting | 5 | Describe the setting, locations, and relevant dates, including periods of recruitment, treatment, follow-up, and data collection | (Methods) Data collection was conducted via an online survey from September 11th to October 2nd, 2025. The recruitment was posted on the intranet of National Office of Investigation. All participants provided informed consent to participate in the study. |
| Participants | 6 | (*a*) *Cohort study*—Give the eligibility criteria, and the sources and methods of selection of participants. Describe methods of follow-up | (Methods) The recruitment was posted on the intranet of National Office of Investigation, ensuring that all participants are FI members of South Korea. Data collection was conducted anonymously via the online survey platform Qualtrics. |

| Variables | 7 | Clearly define all outcomes, treatments, predictors, potential confounders, and effect modifiers. Give diagnostic criteria, if applicable | (Methods; Measures) Demographic and work-related information, Duty types and stressors at site, Psychological Health (PTSD, depression, anxiety, suicide risk, alcohol use) |
| --- | --- | --- | --- |
| Data sources/ measurement | 8* | For each variable of interest, give sources of data and details of methods of assessment (measurement). Describe comparability of assessment methods if there is more than one group |  |
| Bias | 9 | Describe any efforts to address potential sources of bias | (Discussion; Limitation) The reliance on self-report measures and convenient sampling which can be subject to bias within the culture of FIs stigmatizing mental health, could result in underreport of mental health symptoms. |
| Study size | 10 | Explain how the study size was arrived at | (Discussion; Limitation) This study included a nationwide survey sample of FIs in Korea… This stigmatizing culture among the uniformed personnel appears to affect a relatively lower rate of complete survey response (66.8%). |
| Quantitative variables | 11 | Explain how quantitative variables were handled in the analyses. If applicable, describe which groupings were chosen and why | (Methods) Descriptive statistics, including means, standard deviations, and frequencies, were calculated to characterize the study sample in terms of demographics, on-site duties, stressors, and mental health scores… All statistical analyses were conducted using IBM SPSS Statistics (Version 29).  (Results) Table 2 presents the overall mental health status of FIs and prevalence comparisons between engaged and non-deployed FIs. For investigating mental health and influencing key factors among engaged FIs, the indirectly and directly engaged FIs (n = 20 and n = 204, respectively) were combined (engaged FI; n = 224). To justify the consolidation, we conducted sensitivity analyses by comparing fully adjusted logistic regression models (covariates: basic characteristics, MFI stressors or duty types) of engaged FIs (n = 224) and directly engaged FIs (n = 204). Two models showed similar results, especially both of which showed the same significant variable in MFI stressor model (emotional identification with the bereaved; engaged FIs OR = 3.29 [1.14, 9.48]; directly engaged FIs OR = 3.78 [1.22, 11.69]). Detailed results were presented in Supplementary Tables 1 and 2. |
| Statistical methods | 12 | (*a*) Describe all statistical methods, including those used to control for confounding | (Methods) To examine associations between engaged FI experience and clinical outcomes—specifically probable PTSD, depression, anxiety, suicide risk, and alcohol use—two-tailed Chi-square tests were performed. We used Spearman’s correlation analysis to explore correlation between PTSD symptom scores (avoidance, hyperarousal, intrusion, numbing, total) and duty roles or on-site stressors. The significance level (.05) was adjusted for using the Bonferroni correction (5 symptom scores * 17 roles & stressors = 85 tests; α = 0.05/85= 0.00059).  Logistic regression was used to identify on-site risk factors, duty types and stressors … all of which satisfy the traditionally recommended guidelines for logistic regression. |
|  |  | (*b*) Describe any methods used to examine subgroups and interactions |  |
|  |  | (*c*) Explain how missing data were addressed | (Methods) For the logistic regression, we dichotomously coded responses as 1 if participants reported experiencing the corresponding duty type or stressor, and as 0 if they did not experience it or did not participate in the work (non-deployed FIs, n = 511).  (Results) A total of 735 FIs responded to the survey, and there was no missing data.  (Methods) Of the total of 1,101 records, valid responses of 735 participants were analyzed after elimination of the uncompleted or random responses filtered by time stamps and response patterns. |
|  |  | (*d*) *Cross-sectional study*—If applicable, describe analytical methods taking account of sampling strategy | (Methods) Of the total of 1,101 records, valid responses of 735 participants were analyzed after elimination of the uncompleted or random responses filtered by time stamps and response patterns.  (Discussion; Limitation) The reliance on self-report measures and convenient sampling which can be subject to bias within the culture of FIs stigmatizing mental health, could result in underreport of mental health symptoms. This stigmatizing culture among the uniformed personnel appears to affect a relatively lower rate of complete survey response (66.8%). |
|  |  | (*e*) Describe any sensitivity analyses | (Methods) For significant results, post-hoc analyses using standardized residuals were conducted… The significance level (.05) was adjusted for using the Bonferroni correction (5 symptom scores * 17 roles & stressors = 85 tests; α = 0.05/85= 0.00059).  (Results) To justify the consolidation, we conducted sensitivity analyses by comparing fully adjusted logistic regression models (covariates: basic characteristics, MFI stressors or duty types) of engaged FIs (n = 224) and directly engaged FIs (n = 204). Two models showed similar results, especially both of which showed the same significant variable in MFI stressor model (emotional identification with the bereaved; engaged FIs OR = 3.29 [1.14, 9.48]; directly engaged FIs OR = 3.78 [1.22, 11.69]). Detailed results were presented in Supplementary Tables 1 and 2. |

| **Results** |  |  |  |
| --- | --- | --- | --- |
| Participants | 13* | (a) Report numbers of individuals at each stage of study—eg numbers potentially eligible, examined for eligibility, confirmed eligible, included in the study, completing follow-up, and analysed | (Methods) Of the total of 1,101 records, valid responses of 735 participants were analyzed after elimination of the uncompleted or random responses filtered by time stamps and response patterns. |
|  |  | (b) Give reasons for non-participation at each stage |  |
|  |  | (c) Consider use of a flow diagram |  |
| Descriptive data | 14* | (a) Give characteristics of study participants (eg demographic, clinical, social) and information on exposures and potential confounders | (Results) A total of 735 FIs responded to the survey, and their basic characteristics are presented in Table 1. |
|  |  | (b) Indicate number of participants with missing data for each variable of interest | (Methods) Data collection was conducted anonymously via the online survey platform Qualtrics. … Of the total of 1,101 records, valid responses of 735 participants were analyzed after elimination of the uncompleted or random responses filtered by time stamps and response patterns. |
| Outcome data | 15* | *Cross-sectional study—*Report numbers of outcome events or summary measures | (e.g. Results): Our findings revealed that approximately one in ten FIs (10.2%, 95% CI: [8.0, 12.4]) screened positive for probable PTSD, suggesting that a substantial proportion of these professionals experience significant psychological distress. Among those who identified a traumatic event, nearly 60% cited routine occupational incidents—such as homicides and accidents—rather than major disasters. |
| Main results | 16 | (*a*) Give unadjusted estimates and, if applicable, confounder-adjusted estimates and their precision (eg, 95% confidence interval). Make clear which confounders were adjusted for and why they were included | (e.g. Methods) We classified participants as having probable PTSD only if they both indicated a traumatic event and exceeded the IES-R-K cutoff point (25 or over).  (e.g. Table 2; Total FIs) IES-R ≥25: n = 92, 12.5%, 95% CI [10.1, 14.9]  Probable PTSD (≥ 25 & reported traumatic event): n = 75, 10.2%, 95% CI [8.0, 12.4]  (e.g. Results) The logistic regression analyses with experiences of on-site duty types controlling for confounders were conducted (Table 4) … The logistic regression analyses with experiences of on-site stressors in Muan were also conducted (Table 5). |
|  |  | (*b*) Report category boundaries when continuous variables were categorized | (e.g. Table 2; PHQ-9) No depression (0-4); Mild depression (5-9); Moderate depression (10-19); Severe depression (≥20); Probable clinical depression (≥10) |
|  |  | (*c*) If relevant, consider translating estimates of relative risk into absolute risk for a meaningful time period | N/A |
| Other analyses | 17 | Report other analyses done— analyses of subgroups and interactions, and sensitivity analyses | (e.g. Results) Among engaged FIs, two in three (n = 148, 66.1%) nominated their most distressing event at their work, half of which nominated Jeju Air crash (n = 74, 33.0%). The proportion of FIs who reported the most traumatic event among engaged FIs is significantly higher than that of non-deployed FIs (x^2= 33.0,df=1,p< .001), and its effect size is small (Cramer^' s V= .212). Post-hoc analysis using adjusted standardized residuals revealed that on-site group was significantly more likely to report the most traumatic event than expected (z=5.75,p< .01), while non-deployed group was significantly less likely (z=-5.75,p< .01). |
| **Discussion** |  |  |  |
| Key results | 18 | Summarise key results with reference to study objectives | (Discussion) The study succeeded in specifically elucidating which FI duty types and stressors were stressful and associated with higher PTSD vulnerability, including catastrophic exposure, identification-related experience and poor workplace condition. |
| Limitations | 19 | Discuss limitations of the study, taking into account sources of potential bias or imprecision. Discuss both direction and magnitude of any potential bias | (Discussion) This study yet holds several limitations. The reliance on self-report measures and convenient sampling which can be subject to bias within the culture of FIs stigmatizing mental health, could result in underreport of mental health symptoms. This stigmatizing culture among the uniformed personnel appears to affect a relatively lower rate of complete survey response (66.8%) … may affect the duty-related psychological symptoms. |
| Interpretation | 20 | Give a cautious overall interpretation of results considering objectives, limitations, multiplicity of analyses, results from similar studies, and other relevant evidence | (Discussion) The present study has significant implications for mental health of FIs in Korea. We documented that FIs were routinely exposed to traumatic events, therefore highly vulnerable to probable PTSD and mental health conditions. This necessitates a focus on prevention and intervention measures. |
| Generalisability | 21 | Discuss the generalisability (external validity) of the study results | (Conclusion) As this is the first study to explore the mental health of FIs after an MFI, the findings emphasize the importance of mental health screening and psychological services for FIs deployed to disaster sites. Ultimately, it is imperative to provide PTSD-specific evidence-based psychotherapy to FIs, and to be systematically empowered based on the principles of TIC. |
| **Other information** |  |  |  |
| Funding | 22 | Give the source of funding and the role of the funders for the present study and, if applicable, for the original study on which the present article is based | (Funding) This work was supported by the Korean Radio Promotion Association and the National Research Foundation of Korea (2023S1A5C2A07095987). |

*Give information separately for exposed and unexposed groups.

**Note**: An Explanation and Elaboration article discusses each checklist item and gives methodological background and published examples of transparent reporting. The STROBE checklist is best used in conjunction with this article (freely available on the Web sites of PLoS Medicine at http://www.plosmedicine.org/, Annals of Internal Medicine at http://www.annals.org/, and Epidemiology at http://www.epidem.com/). Information on the STROBE Initiative is available at www.strobe-statement.org.
