# Supplementary material for: The psychological health of forensic investigators following mass fatality incidents: a cross-sectional study 9 months after the Jeju Air crash in South Korea
Source: Front Public Health. 2026 May 29;14:1819655. doi: 10.3389/fpubh.2026.1819655 (PMC13259849; doi:10.3389/fpubh.2026.1819655)
Supplement: Supplementary file 2 [file Data_sheet_2.docx]

**Supplementary Table 1** Logistic analysis of associated MFI duty types with probable PTSD among engaged FIs (n = 224) and directly engaged FIs (n = 204)

| **Predictors** | | **Probable PTSD (Odds Ratio [95% CI])** | |  |
| --- | --- | --- | --- | --- |
|  |  | **All Adjusted**  *(Covariates: baseline characteristics, all duty types)* | |  |
|  |  | **Engaged FIs**  n = 224  34 events (Probable PTSD)  12 predictors | **Directly engaged FIs**  n = 204  34 events (Probable PTSD)  12 predictors |  |
| **Step 1.  Baseline characteristics** | Sex | 0.60 [0.22, 1.66] | 0.48 [0.16, 1.41] |  |
|  |  |  |  |  |
|  | Age | 1.02 [0.95, 1.10] | 1.02 [0.95, 1.10] |  |
|  | Service year | 1.07 [0.98, 1.16] | 1.06 [0.98, 1.16] |  |
|  | 5+ exposures to severely damaged bodies | 2.65 [0.55, 12.83] | 3.16 [0.63, 15.86] |  |
|  | 5+ exposures to massive damaged bodies | 3.02 [1.11, 8.21] * | 2.70 [0.98, 7.39] |  |
| **Step 2.  MFI**  **Duty types** | on-site recovery | 0.51 [0.21, 1.28] | 0.40 [0.16, 1.00] |  |
|  | body classification | 2.29 [0.71, 7.43] | 1.86 [0.58, 5.98] |  |
|  | autopsy | 1.00 [0.38, 2.67] | 0.84 [0.31, 2.25] |  |
|  | body reconstruction | 2.33 [0.90, 6.08] | 2.12 [0.80, 5.65] |  |
|  | collection of samples from bereaved family members | 2.19 [0.64, 7.52] | 2.72 [0.73, 10.06] |  |
|  | release of identified remains to families | 0.62 [0.23, 1.64] | 0.63 [0.23, 1.68] |  |
|  | administrative support | 1.35 [0.47, 3.86] | 1.48 [0.52, 4.27] |  |
| $R^{2}$ | | 0.246 | 0.255 |  |
|  |  |  |  |  |
| $x^{2}/df$ | | 34.0/12*** | 33.5/12*** |  |

*Note. *p < .05, **p < .01, *** p < .001*

**Supplementary Table 2** Logistic analysis of associated MFI stressors with probable PTSD among engaged FIs (n = 224) and directly engaged FIs (n = 204)

| **Predictors** | | **Probable PTSD (Odds Ratio [95% CI])** | |  |
| --- | --- | --- | --- | --- |
|  |  | **All Adjusted**  *(Covariates: baseline characteristics, all duty types)* | |  |
|  |  | **Engaged FIs**  n = 224  34 events (Probable PTSD)  15 predictors | **Directly engaged FIs**  n = 204  34 events (Probable PTSD)  15 predictors |  |
| **Step 1.  Baseline characteristics** | Sex | 0.72 [0.25, 2.12] | 0.63 [0.21, 1.88] |  |
|  |  |  |  |  |
|  | Age | 1.00 [0.93, 1.08] | 1.00 [0.93, 1.08] |  |
|  | Service year | 1.08 [0.99, 1.18] | 1.07 [0.98, 1.17] |  |
|  | 5+ exposures to severely damaged bodies | 2.99 [0.55, 16.21] | 3.74 [0.66, 21.30] |  |
|  | 5+ exposures to massive damaged bodies | 2.37 [0.87, 6.45] | 2.20 [0.81, 5.97] |  |
| **Step 2.  MFI**  **Stressors** | exposure to (severely) damaged human remains | 0.43 [0.08, 2.22] | 0.33 [0.06, 1.82] |  |
|  | exposure to a massive number of human remains | 0.98 [0.24, 3.89] | 0.63 [0.15, 2.62] |  |
|  | exposure to infant or child remains | 1.33 [0.51, 3.42] | 1.32 [0.51, 3.41] |  |
|  | (distressing) odors | 1.07 [0.41, 2.82] | 1.03 [0.39, 2.72] |  |
|  | emotional identification with the victim | 1.10 [0.42, 2.87] | 1.18 [0.44, 3.19] |  |
|  | emotional identification with the bereaved | 3.29 [1.14, 9.48]* | 3.78 [1.22, 11.69]* |  |
|  | excessive workload | 1.28 [0.45, 3.66] | 1.32 [0.45, 3.89] |  |
|  | poor working conditions | 1.06 [0.32, 3.57] | 0.94 [0.28, 3.15] |  |
|  | pressure of making mistakes | 1.76 [0.66, 4.67] | 1.68 [0.63, 4.50] |  |
|  | unorganized system and other administrative system-related stressors | 0.93 [0.36, 2.38] | 0.98 [0.37, 2.56] |  |
| $R^{2}$ | | 0.196 | 0.193 |  |
|  |  |  |  |  |
| $x^{2}/df$ | | 37.3/15*** | 35.5/15*** |  |

*Note. *p < .05, **p < .01, *** p < .001*
